# Translocating the blood-brain barrier using electrostatics

**DOI:** 10.3389/fncel.2012.00044

**Published:** 2012-10-11

**Authors:** Marta M. B. Ribeiro, Marco M. Domingues, João M. Freire, Nuno C. Santos, Miguel A. R. B. Castanho

**Affiliations:** Instituto de Medicina Molecular, Faculdade de Medicina da Universidade de LisboaLisboa, Portugal

**Keywords:** blood-brain barrier, drug targeting, blood cells, cell surface charge, zeta-potential

## Abstract

Mammalian cell membranes regulate homeostasis, protein activity, and cell signaling. The charge at the membrane surface has been correlated with these key events. Although mammalian cells are known to be slightly anionic, quantitative information on the membrane charge and the importance of electrostatic interactions in pharmacokinetics and pharmacodynamics remain elusive. Recently, we reported for the first time that brain endothelial cells (EC) are more negatively charged than human umbilical cord cells, using zeta-potential measurements by dynamic light scattering. Here, we hypothesize that anionicity is a key feature of the blood-brain barrier (BBB) and contributes to select which compounds cross into the brain. For the sake of comparison, we also studied the membrane surface charge of blood components—red blood cells (RBC), platelets, and peripheral blood mononuclear cells (PBMC). To further quantitatively correlate the negative zeta-potential values with membrane charge density, model membranes with different percentages of anionic lipids were also evaluated. From all the cells tested, brain cell membranes are the most anionic and those having their lipids mostly exposed, which explains why lipophilic cationic compounds are more prone to cross the blood-brain barrier.

## Introduction

Membranes are not considered anymore as mere barriers limiting the cellular content. An increased complexity of membrane composition, dynamics and functionality has been recognized in recent years. Membrane surface charge, which is the cumulative effect of charged proteins, ions, and lipid headgroups on the membrane, seems to play an essential role in this functionality, with a crucial part in homeostasis and protein targeting, among other processes (Goldenberg and Steinberg, 2010).

Mammalian cell membranes are generally known to be anionic, but a comprehensive study of their charge density has never been performed. First reports and concerns about the charge of cells appeared in the 1960's. Most of these initial studies evaluated the electrophoretic mobility and were performed for red blood cells (RBC). Elul (1967) was the first to observe that the negative electrostatic charge of RBC is located at fixed positions on the cell surface. Such negative charge was mainly attributed to the presence of sialic acid residues at the membrane level (Eylar et al., 1962). These authors also reported that no significant differences existed between species and cell types.

In the last decades, the development of the biological and biomedical applications of dynamic light scattering approaches brought new insights to this field. In the absence of a direct measurement of the surface charge, zeta-potential has proven to be very useful. Using this technique, some authors showed that RBC from human diabetic patients exhibited lower zeta-potential values than RBC from healthy donors (Adak et al., 2008). A correlation between increased zeta-potential and aging of RBC was also reported (Chen et al., 2007; Carvalho et al., 2011). As the dependence of hematology unbalance on alterations in membrane surface charge of cells became evident, an increased interest in the interaction between vascular endothelial cells (EC) and blood components emerged. Moreover, vascular permeability to macromolecules seemed to be dependent not only on molecular size but also on the electrostatic charge (Klein et al., 1992). As a matter of fact, many studies show that cationic compounds have an increased tendency to translocate cellular membranes, relative to anionic compounds. A clear example resides on the cell penetrating peptides, a cationic class of compounds that have been successfully used for drug delivery into mammalian cells (Henriques et al., 2006).

We recently characterized the membrane surface charge of EC from two different origins: bovine brain capillary endothelial cells (BCEC) and human umbilical vascular endothelial cells (HUVEC), and their interaction with analgesic peptides through zeta-potential measurements (Ribeiro et al., 2011). The use of human primary brain cells is hampered both by ethical and technical issues, and immortalized cell lines display an elevated expression of phosphatidylserine (PS) in the outer leaflet of the cell membrane when compared to normal cells, and therefore, would not provide a truthful zeta-potential value (Zwaal et al., 2005). For these reasons, we decided to use bovine cells due to brain size, availability, performance, and strong correlation with human *in vivo* data (Gumbleton and Audus, 2001; Garberg et al., 2005). BCEC membranes revealed to be more anionic than HUVEC membranes. In addition, BCEC showed a preferential interaction with a peptide that combined cationic and lipophilic characteristics. Now, we hypothesize that anionicity is a distinctive feature of brain endothelium. Confirmation or rebuttal of such hypothesis requires a comparative study of the anionicity of the cells' membranes that are exposed to drugs during their systemic distribution along the bloodstream—RBC, platelets, and leukocytes, such as peripheral blood mononuclear cells (PBMC). In addition, several drugs are incorporated in the RBC, largely reducing their bioavailability and further reinforcing the need to map the factors that govern the interaction of drugs with the cell membranes they contact with.

In the present work, we used zeta-potential measurements by dynamic light scattering to quantify the membrane surface charge of the main blood cellular components: RBC, platelets, and PBMC, and compared these with the values previously obtained for BCEC and HUVEC. For the sake of simplicity, we also performed experiments with membrane model systems so that an intuitive anionicity scale can be used as a working tool. Our results show that BCEC possess the most negatively charged membrane. All blood components display similar zeta-potential values and these do not differ from HUVEC membranes, suggesting that electrostatic repulse between vascular endothelium and blood components is crucial for maintaining homeostasis.

## Materials and methods

### Blood

Human blood samples were obtained from adult healthy volunteers, at the public blood bank Instituto Português do Sangue (IPS, Lisbon, Portugal), with their previous informed written consent, under an institutional agreement between IPS and the Instituto de Bioquímica of the Faculdade de Medicina da Universidade de Lisboa (FMUL). This study was approved by the FMUL Ethics Committee. Blood was collected into K_3_EDTA anticoagulant tubes (Vacuette, Greiner Bio-one, Kremsmünster, Austria).

### Blood components isolation

Total erythrocyte population was separated from the other blood components by centrifugation at 200 *g* for 10 min, at room temperature. Erythrocytes were washed three times with 4-(2-hydroxyethyl)-1-piperazineethanesulfonic acid (HEPES) buffer, pH 7.4, and centrifuged at 2000 *g* for 10 min. This process was repeated twice. Packed erythrocytes were re-suspended in the same buffer and diluted for a final hematocrit of 0.0035%, corresponding approximately to 4 × 10^8^ RBC/L. Zeta-potential measurements were performed with 4 × 10^5^ RBC/mL in HEPES buffer.

Platelets were isolated from platelet-rich plasma (PRP). Briefly, PRP was separated by centrifugations at 220 *g* for 7 min, at 10°C. Platelets were pelleted from PRP at 1620 *g* for 10 min, at 10°C, and washed three times with HEPES buffer. The final pellet was re-suspended in 1 mL and counted. Zeta-potential measurements were conducted with 4 × 10^6^ platelets/mL in HEPES buffer.

PBMC were isolated by density gradient using Ficoll-Paque Plus (GE Healthcare, Little Chalfont, UK), accordingly to manufacturer's instructions. Cells were counted at the microscope with trypan blue and were used for zeta-potential measurements at 4 × 10^5^ cells/mL in HEPES buffer.

### Lipid vesicles preparation

Large unilamellar vesicles (LUVs) with approximately 100 nm diameter were used. For LUVs preparation, lipids were dissolved in chloroform and the solvent was removed to yield a lipid film. After addition of buffer, hydrated lipid sheets detach spontaneously. Upon agitation and freeze-thaw cycles, a suspension of multilamellar vesicles was obtained and further extruded to produce LUVs (Mayer et al., 1986). LUVs have a lipid packing close to planar membrane due to their larger radii of curvature relative to molecular dimensions and are more stable than other vesicles, making them ideal mammalian membrane models. Also, LUVs inner and outer layers display similar superficial areas (Ribeiro et al., 2010). These vesicles can be easily produced with different biologically relevant lipid compositions (e.g., presence of charged phospholipids or different cholesterol content), accounting for the contributions of hydrophobic and electrostatic interactions, membrane fluidity, and phase separation, or can even be prepared directly from lipids extracted from a certain type of cell. Here, systems constituted by POPC (1-palmitoyl-2-oleyol-sn-glycero-3-phosphocoline) and POPG {1-palmitoyl-2-oleyl-sn-glycero-3-[phosphor-rac-(1-glycerol)]} were studied with the following proportions: 95:5, 90:10, and 85:15. Zeta-potential measurements were conducted with a lipid composition of 200 μM, in phosphate buffered saline (PBS, pH 7.4). LUVs constituted by pure POPC or POPC:POPG 80:20, 60:40, and 40:60 were also evaluated to characterize the relationship between zeta-potential values and the total anionic percentage of lipid in the membrane bilayer.

### Zeta-potential measurements

Measurements were conducted on a dynamic light scattering and zeta-potential Malvern Zetasizer Nano ZS equipment (Malvern, UK), with a He-Ne laser (λ = 632.8 nm). The zeta-potential (ζ) of the samples was determined, at 25°C, from the mean of a minimum of 15 measurements (60 runs each), with 90 s in between measurements, with an applied potential of 40 V, using disposable zeta-potential cells with platinum gold-coated electrodes (Malvern, UK). Samples were incubated in the cell holder for 15 min prior to measurement. 1-[4-(Trimethylamino)phenyl]-6-phenyl-1,3,5-hexatriene (TMA-DPH, Invitrogen) was added for a final concentration of 54 μM and incubated for 30 min previous to measurements to allow complete equilibration of TMA-DPH with cells or LUVs. Each represented group value is an average of at least two independent measurements. The electrophoretic mobility obtained was used for the zeta-potential calculation through the Smoluchowski equation (Domingues et al., 2008).

(1) $$\zeta =\frac{4\pi \eta \mu }{\varepsilon }$$

where μ represents the electrophoretic mobility, η the viscosity of the solvent and ε its dielectric constant. The viscosity of all tested samples was similar, despite the differences in the cells' concentration used; turbidity was kept below the threshold that significantly attenuates the incident light intensity in the sample and samples were prepared in the same buffer (Anderson, 1981; Castanho et al., 1997; Delgado et al., 2005; Kaszuba et al., 2010). Therefore, in the experimental conditions used, the slight concentration differences between samples evaluated do not influence zeta-potential measurements.

### Statistical analysis

Data are expressed as mean ± standard error of the mean (SEM). Each group value is an average of at least two independent measurements. The significance of differences in each group between control and TMA-DPH treated cells/vesicles was evaluated by Student's *t*-test. The significance of differences between cell types/vesicles with distinct composition was analyzed with One-Way ANOVA followed by Bonferroni's multiple comparison test. All statistical analyses were calculated with Prism software (GraphPad Software, version 5).

## Results

### Zeta-potential of blood components

Although there are a few reports regarding the membrane surface charge of some RBC populations, a simultaneous approach to all blood components in the same experimental conditions to the best of our knowledge has never been performed. To characterize the membrane surface charge of RBC, platelets, and PBMC, these constituents were isolated and their zeta-potential values measured (Table 1).

**Table 1:** **Zeta-potential of blood components, endothelial cells, and membrane model systems constituted by POPC and POPG**.

		**ζ (mV)**	**Δζ (mV)**
**BLOOD COMPONENTS**			
RBC	Buffer	−10.80 ± 1.63	13.17 ± 2.42
	TMA-DPH	2.37 ± 0.79	
Platelets	Buffer	−10.75 ± 1.17	1.43 ± 2.12
	TMA-DPH	−9.33 ± 0.96	
PBMC	Buffer	−11.95 ± 0.66	1.25 ± 1.79
	TMA-DPH	−10.70 ± 1.13	
**ENDOTHELIAL CELLS**			
BCEC	Buffer	−15.28 ± 0.58	3.15 ± 1.07
	TMA-DPH	−12.13 ± 0.49	
HUVEC	Buffer	−12.89 ± 0.56	1.74 ± 1.05
	TMA-DPH	−11.15 ± 0.49	
**LUVs**			
5% POPG	Buffer	−7.06 ± 1.33	12.87 ± 3.35
	TMA-DPH	5.82 ± 2.02	
10% POPG	Buffer	−12.60 ± 0.63	9.88 ± 1.50
	TMA-DPH	−2.73 ± 0.87	
15% POPG	Buffer	−19.80 ± 0.66	11.59 ± 2.16
	TMA-DPH	−8.21 ± 1.50	

Cells or LUVs were incubated with TMA-DPH for a final concentration of 54 μM at 25°C and zeta-potential was measured. RBC (4 × 10^5^ cells/mL), platelets (4 × 10^6^ platelets/mL), PBMC (4 × 10^5^ cells/mL), HUVEC and BCEC (Ribeiro et al., 2011), and LUVs (200 μM) concentrations were kept constant. Data shown as mean ± SEM; each group value is an average of at least two independent measurements.

**Figure 1:**
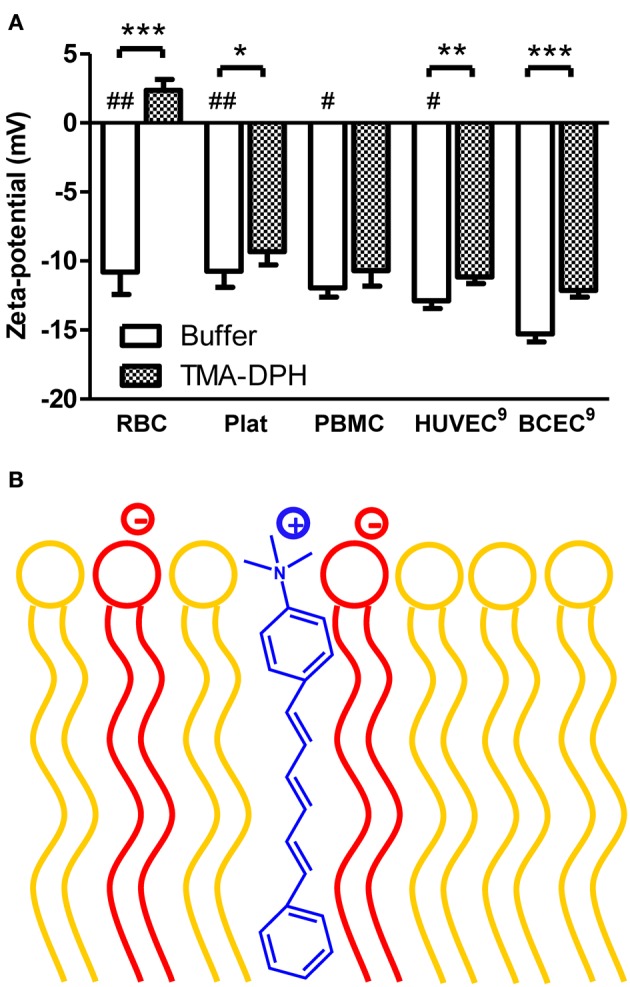
**Zeta-potential of blood components and endothelial cells of mammals in the absence and in the presence of TMA-DPH. (A)** RBC (4 × 10^5^ cells/mL), platelets (4 × 10^6^ platelets/mL), and PBMC (4 × 10^5^ cells/mL), HUVEC and BCEC (1 × 10^5^ cells/mL) were incubated with TMA-DPH (54 μM) at 25°C and zeta-potential was measured. Data shown as mean ± SEM; each group value is an average of at least two independent measurements. ^*^P < 0.05; ^**^P < 0.01; ^***^P < 0.001 vs. unlabeled samples, *t*-test; and ^#^*P* < 0.05, ^##^*P* < 0.01 vs. BCEC, One-Way ANOVA, Bonferroni's multiple comparison test. **(B)** Schematic representation of TMA-DPH localization in the lipid membrane. The cationic trimethylamino group of TMA-DPH (in blue) locates near the polar heads of phospholipids; anionic phospholipids are represented in red and zwitterionic in yellow.

As shown in Figure 1A, all blood components display similar zeta-potential values, between −10.75 mV (platelets) and −11.95 mV (PBMC). Furthermore, zeta-potential of HUVEC (Ribeiro et al., 2011) is within the same range of values as RBC, platelets, and PBMC. The similar charge between blood components and vascular endothelium causes a constant repulsion between these cells.

To test the ability of zeta-potential to report electrostatic interactions in the cells' surfaces, we used as a positive control TMA-DPH, a probe of membrane dynamics in living cells. It is a cationic probe (+1), due to its –NH^+^_3_ moiety that intercalates at the level of hydrophilic head groups of membrane phospholipids, increasing the electrostatic charge of the outer layer of the cell membrane (Figure 1B). Upon interaction, TMA-DPH is expected to contribute to reduce anionicity of the cell membrane. After incubation with TMA-DPH, only PBMC did not present a significant change on the zeta-potential value (Figure 1A). PBMC have a high immune activity, constantly recognizing and destroying exogenous pathogens. These defense mechanisms may explain the lack of differences between unlabeled and TMA-DPH treated PBMC. On the other hand, TMA-DPH incubation with RBC resulted in the higher difference observed between control and TMA-DPH treated cells. The extent of this variation can be appraised through Δζ = ζ_TMA–DPH_ − ζ_buffer_ (Table 1). In fact, ζ of RBC changed from negative (−10.80 ± 1.634 mV, buffer) to positive values (2.37 ± 0.787 mV, 54 μM TMA-DPH), resulting in Δζ = 13.17 ± 2.42 mV. This considerable alteration in the zeta-potential, which can be understood in terms of TMA-DPH affinity toward the RBC membrane, indicates an entrapment of the probe beyond electrostatic equivalence.

When put together, these results show that from all the cells tested, BCEC display the most negative membrane charge. In addition, BCEC interaction with TMA-DPH was also extremely significant (*P* < 0.001), which indicates that zeta-potential is an adequate technique to assess the membrane charge of these cells and to report the interaction with cationic molecules.

### Zeta-potential of LUVs

**Figure 2:**
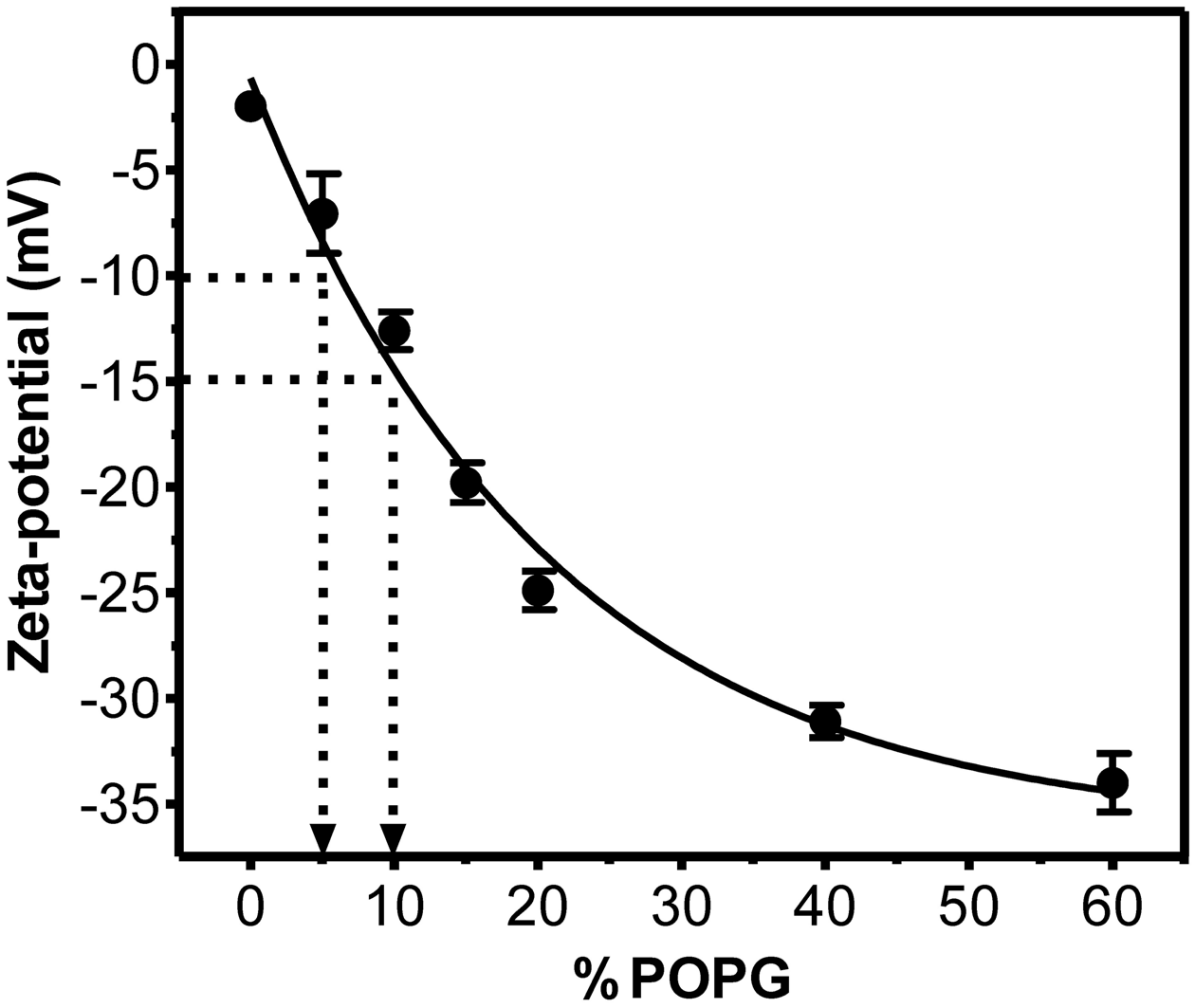
**Relation between zeta-potential and fraction of negatively charged phospholipids in LUVs constituted by POPC and POPG.** LUVs (200 μM) were prepared in PBS buffer and zeta-potential measured at 25°C. Each group value is an average of at least two independent measurements; data shown as mean ± SEM. Solid line represents an exponential function fit to the data. It is noteworthy that a change from ζ = −10 mV to ζ = −15 mV (corresponding to a change from RBC/PBMC/platelets to BCEC) is equivalent to a 2-fold increase in surface charge density (inserted arrows).

**Figure 3:**
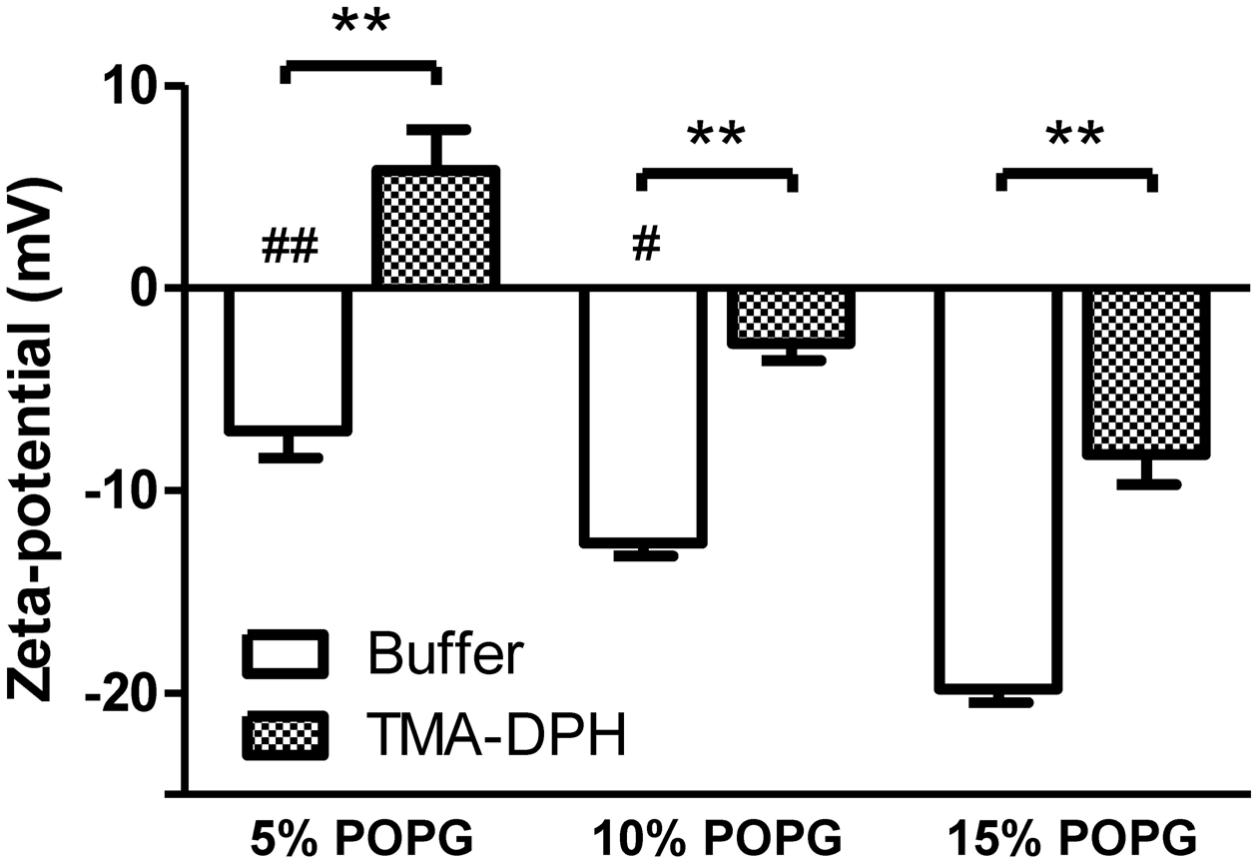
**Zeta-potential of membrane model systems of POPC and POPG.** LUVs were incubated with TMA-DPH (54 μM) at 25°C and zeta-potential was measured. Lipid concentration was kept constant at 200 μM. Data shown as mean ± SEM; each group value is an average of at least two independent measurements. ^**^P < 0.01 vs. unlabeled samples, *t*-test; and ^#^*P* < 0.05, ^##^*P* < 0.01 vs. 15% POPG, One-Way ANOVA, Bonferroni's multiple comparison test.

To have a semi-quantitative correlation between ζ and membrane charge, we used cell membrane model systems—LUVs—and measured their zeta-potential values. Mixtures of fluid zwitterionic POPC with anionic POPG (up to 60%) were used. Electrostatic interactions are expected to be independent from the nature of the anionic lipid (Buser et al., 1994). As expected, increased POPG concentration in the membrane resulted in more negative zeta-potential values (Figures 2 and 3). Figure 2 shows that a drop in ζ from −10 mV (RBC, PBMC, platelets, HUVEC) to −15 mV corresponds to an apparent change in anionic net surface charge density of roughly 2-fold, which is extremely significant when interaction with cationic drugs is considered.

Incubation with TMA-DPH (Figure 3) promoted the alteration of zeta-potential toward neutralization and in the case of 5% POPG overcompensation to positive values (from −7.06 mV to 5.82 mV). The Δζ values show that TMA-DPH has the same effect on all tested lipidic mixtures (Table 1). This interaction profile is also perceived in the statistical test applied for ζ_buffer_ vs. ζ_TMA–DPH_ for all lipid mixtures (*P* < 0.001, Figure 3).

## Discussion

Recent developments at the interface of cell biology and biophysics have established a new paradigm in protein targeting and function, in which the electrostatic charge at the surface of a biological membrane has emerged as a key factor (Yeung et al., 2008, 2009; Goldenberg and Steinberg, 2010). In the specific case of brain targeted compounds, the charge at the surface of brain endothelium appears to have a role (dos Santos et al., 1995). However, non-specific interactions are often disregarded in biomedical investigation and a comprehensive study on membrane surface charge of different mammalian cells remains elusive. In this study, we quantified the membrane surface charge of blood components and compared it with the values obtained for EC from two different origins—brain and umbilical cord—through dynamic light scattering based methodologies.

The human RBC membrane has served over the years as a prototypical model for the structure of the eukaryotic plasma membrane and therefore it is by far the most studied cell membrane (Daleke, 2008). This justifies that the majority of zeta-potential measurements by dynamic light scattering studies with mammalian cells were performed with RBC. The values reported here for RBC are within the ranges published for two erythrocytes' populations: young and old (Chen et al., 2007; Carvalho et al., 2011). The considerable effect of TMA-DPH in the zeta-potential value of these cells suggests that RBC may reduce the bioavailability of cationic drugs. Regarding platelets, Tatsumi and colleagues measured ζ = −14 ± 1.64 mV (Tatsumi et al., 1992), which is similar to the value we obtained, despite the fact that different experimental conditions were used. Our results suggest that, in a healthy person, the similarity of membrane charge of blood components and vascular endothelium wall prevents aggregation and thrombus formation and that this may be an essential requirement for homeostasis maintenance.

### Membrane surface charge and phospholipidic composition: is there a correlation?

**Figure 4:**
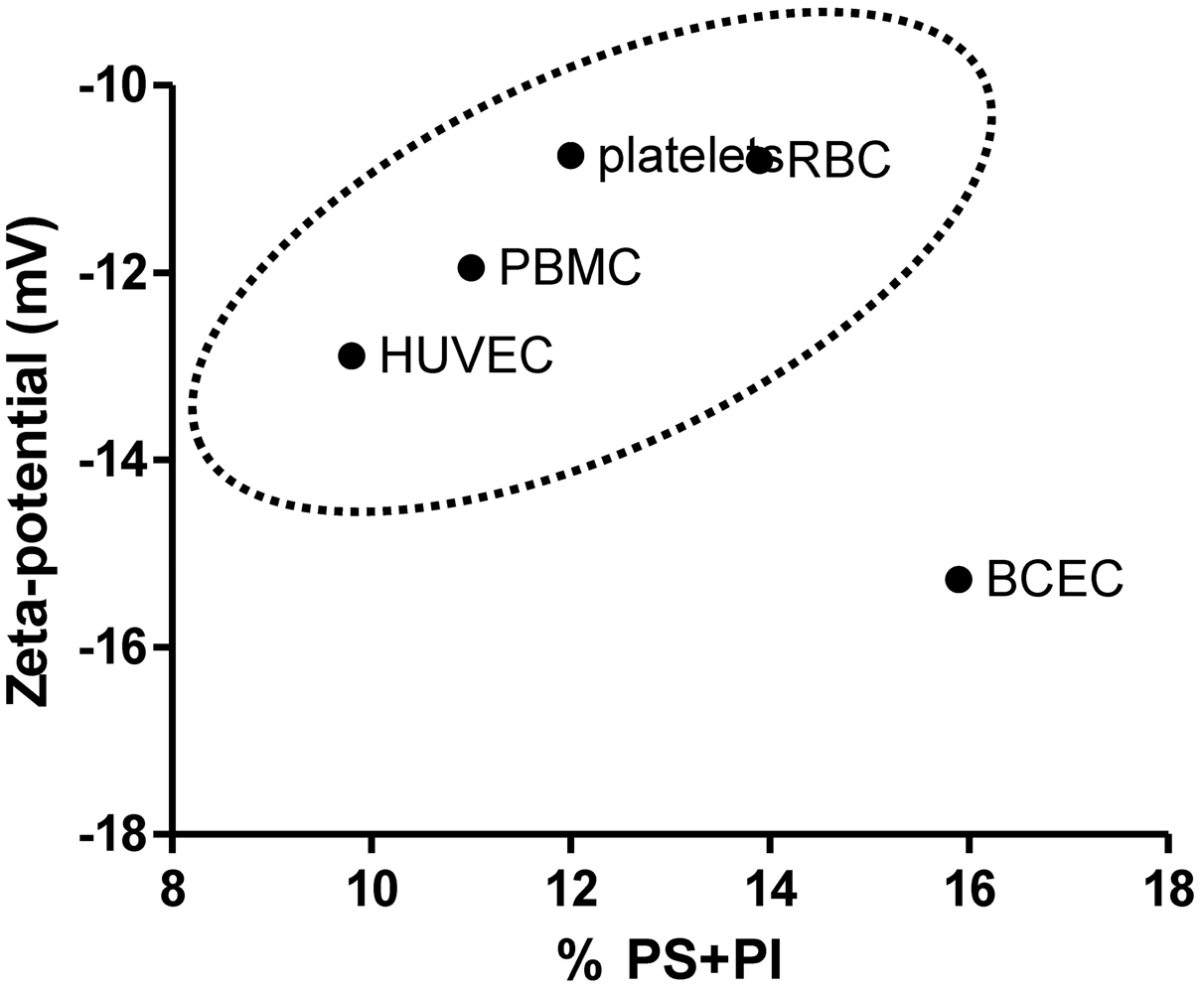
**Correlation between the total percentage of negatively charged phospholipids (PS + PI) in the cells membrane and zeta-potential value.** For PBMC only PS was accounted for (Table 2). BCEC are a particular case where the high percentage of anionic lipids has a strongly negative zeta-potential value as a counterpart.

The charge of plasma cell membrane is influenced, among other factors, by the lipid headgroups in the membrane, especially those that display anionic charge at physiologic pH, such as PS and phosphatidylinositol (PI). The zeta-potential values obtained for cells were correlated with the proportion of negatively charged lipids in the membrane of vesicles. If the main determinant of membrane surface charge was the lipidic composition, a direct correlation between these values and the phospholipid compositions of RBC (Owen et al., 1981), platelets (Owen et al., 1981), PBMC (Kuliszkiewicz-Janus et al., 2005), BCEC (Tewes and Galla, 2001), and HUVEC (Cansell et al., 1997) could be established. Values found in the literature for phospholipid compositions of these cells are summarized in Table 2. The sum of the fractions of anionic lipids (PS + PI) is different between tested cells, and the higher values were found for BCEC. This explain in part why EC from the blood-brain barrier (BBB) have the more negative membrane charge from all the cells tested but there is no direct correlation between the percentage of anionic lipids present in the plasma membrane in the different cells tested and zeta-potential values (Figure 4). Figure 4 shows that the percentage of negatively charged lipids in the membranes of cells does not encompass the anionic net charge density at their surface, BCEC being an exception. This result can be assigned to: (1) Cell membrane asymmetry in terms of lipid composition—the outer layer of RBC, for instance, has only a small percentage of anionic lipids, while the inner layer is much richer (Daleke, 2008), thus reducing the surface charge density of the RBC. (2) Contribution of components other than lipids, such as glycolipids, glycoproteins, and proteins, to surface charge density; this may be the case for HUVEC as there is a relatively high negative zeta-potential value for a small fraction of anionic lipids.

**Table 2:** **Membrane phospholipid composition of blood components—RBC (Owen et al., 1981), platelets (Owen et al., 1981) and PBMC (Kuliszkiewicz-Janus et al., 2005)—and endothelial cells—BCEC (Tewes and Galla, 2001) and HUVEC (Cansell et al., 1997)**.

**Phospholipid**	**Blood components**			**Endothelial cells**	
	**RBC**	**Platelets**	**PBMC**	**BCEC**	**HUVEC**
Phosphatidylcholine (PC)	30.3	40.6	43.4	37.1	53.9
Sphingomyelin (SM)	27.3	19.3	16.9	17.3	22.5
Phosphatidylserine (PS)	13.4	9.3	11.0	8.8	3.0
Phosphatidylinositol (PI)	0.5	2.7	28.7	7.1	6.8
Phosphatidylethanolamine (PE)	28.4	27.9		29.5	13.7

Values shown in percentage, taking into consideration that the sum of PC, SM, PS, PI, and PE proportions represents 100%.

BCEC are a very peculiar case, as the lipid composition seems to suffice to explain their anionicity, which is in agreement with their function: a highly impermeable barrier with low density of receptors. Heavy anionicity and lipid exposition contribute to impermeability (less binding of glycidic or protein groups) and prevent unspecific binding or adhesion of blood components which is significant to prevent thrombus formation. At the same time, these characteristics reveal a specific property of the BBB that is important for drug-targeting: lipophilic cationic drugs are prone to interact with it. Data published by other groups further support our conclusions. dos Santos et al. (dos Santos et al., 1995) found that the higher density of heparin sulfate of BCEC is located at the intercellular junctions, not the cell surface. There are reports of successful drug brain targeting when cationic compounds are linked to the drug (Tamai et al., 1997). Leukocyte adhesion and transendothelial migration, both in the normal state and in inflammatory conditions, were reported to be lower in BCEC in comparison with other EC (Male et al., 1990; Pryce et al., 1994), which is in accordance with the anionicity of BCEC membrane surface.

Compared to blood components (RBC, PBMC, and platelets) and other EC (HUVEC), BCEC have a higher density of anionic surface charges and a higher exposition of “naked” lipid surface. This is a distinctive feature of cell membranes at the BBB level and contributes to its high selectivity. The results reported here explain why lipophilic cationic molecules are prone to interact with the BBB and justify the low adhesion of cells at the brain level.

### Conflict of interest statement

The authors declare that the research was conducted in the absence of any commercial or financial relationships that could be construed as a potential conflict of interest.

## Abbreviations

RBC: red blood cells
EC: endothelial cells
BCEC: brain capillary endothelial cells
HUVEC: human umbilical vein endothelial cells
TMA-DPH: 1-[4-(trimethylamino)phenyl]-6-phenyl-1,3,5-hexatriene
PBMC: peripheral blood mononuclear cells
HEPES: 4-(2-hydroxyethyl)-1-piperazineethanesulfonic acid
PRP: platelet-rich plasma
LUVs: large unilamellar vesicles
POPC: 1-palmitoyl-2-oleyol-sn-glycero-3-phosphocoline
POPG: 1-palmitoyl-2-oleyl-sn-glycero-3-[phosphor-rac-(1-glycerol)]
PBS: phosphate buffered saline
SEM: standard error of the mean
BBB: blood brain barrier
PS: phosphatidylserine, PI, phosphatidylinositol
PC: phosphatidylcholine
SM: sphingomyelin
PE: phosphastidylethanolamine.
